# Guideline concordant therapy improves survival in high-grade endometrial cancer patients

**DOI:** 10.1007/s00432-022-04318-1

**Published:** 2022-10-14

**Authors:** Sophia Scharl, Tim Sprötge, Michael Gerken, Anton Scharl, Olaf Ortmann, Oliver Kölbl, Monika Klinkhammer‑Schalke, Thomas Papathemelis

**Affiliations:** 1 Department of Radiation Oncology, University Hospital Ulm, Ulm, Germany; 2 Tumor Center, Institute for Quality Management and Health Services Research, University of Regensburg, Regensburg, Germany; 3 Department of Gynecology and Obstetrics, University Medical Center Regensburg, Regensburg, Germany; 4 Department of Gynecology and Obstetrics, Klinikum St. Marien Amberg, Amberg, Germany; 5 Department of Radiation Oncology, University Medical Centre Regensburg, Regensburg, Germany; 6 Oncology Competence Center, Klinik Bad Trissl, Oberaudorf, Germany

**Keywords:** High grade, Endometrial cancer, Guideline concordant therapy, S3 guideline

## Abstract

**Purpose:**

Data from randomized controlled trials in high-grade endometrial cancer are scarce due to its low prevalence. Therefore, guideline recommendations in this cancer subtype rely on relatively few randomized trials and data from retrospective studies. The aim of this study was to evaluate the benefits from guideline-concordant therapy in high-grade endometrial cancer in a real-world patient group.

**Methods:**

The effect of treatment according to German S3 guidelines and the former S2k guideline on overall survival (OS) and recurrence-free survival (RFS) was evaluated in a cohort of 293 high-grade endometrial cancer patients.

**Results:**

Treatment concordant with the S3 guideline significantly improved OS (HR 0.623, CI 0.420–0.923, *p* = 0.018) and RFS (HR 0.578, CI 0.387–0.863, *p* = 0.007). Treatment concordant with the S2k guideline did not result in a significantly higher OS (HR 0.783, CI 0.465–1.316, *p* = 0.335) or RFS (HR 0.741, CI 0.347–1.740, *p* = 0.242).

**Conclusion:**

Therapy according to the German S3 guideline improved OS and RFS in univariate as well as multivariate analysis in this cohort of high-grade endometrial cancer patients.

## Introduction

Data from randomized controlled trials in high-grade endometrial cancer are scarce due to its low prevalence. While the PORTEC-3 trial and the NRG/GOG 249 study shed light upon the role of adjuvant therapy in high-risk endometrial cancer, many questions on its treatment remain (de Boer et al. 2018; Randall et al. 2019).

Whether lymphadenectomy improves survival has been studied in a randomized trial with a cohort of predominantly low-risk patients which showed no improvement of survival. For high-risk endometrial cancer, the STATEC trial examining the benefit of lymphadenectomy on survival in stage 1 endometrial cancer and its effect on adjuvant therapy has finished accrual (Mould et al. 2018). Until its publication, the effect of lymphadenectomy in early stage endometrial cancer remains unclear.

Therefore, the guidelines for endometrial cancer are well founded for the more common type 1 cancer. Even though a number of clinical trials have been published on type 2 cancer and high-grade type 1 cancer, experts still have to rely on relatively few randomized trials and data from retrospective studies.

The recommendations certainly changed over the years and different countries recommend slightly different approaches [Kommission Uterus der Arbeitsgemeinschaft Gynäkologische Onkologie e.V. in der Deutschen Gesellschaft für Gynäkologie und Geburtshilfe e.V. sowie in der Deutschen Krebsgesellschaft e. V. 2010; Leitlinienprogramm Onkologie (Deutsche Krebsgesellschaft, Deutsche Krebshilfe, and AWMF) 2018; National Comprehensive Cancer Network 2022]. In 2008, the German Cancer Society issued an S2k-guideline which was considered valid until 2013. Only in 2018, the S2k Guideline was followed by an S3 guideline. Hysterectomy combined with bilateral salpingo-oophorectomy is the cornerstone of surgical treatment in endometrial cancer. Wide-ranging agreement is reached recommending lymphadenectomy in patients with high-grade histology, although it has not yet been confirmed as a curative factor. Whether adjuvant chemotherapy, radiotherapy, or combined radiochemotherapy is recommended depends on histology and stage [Deutsche Krebsgesellschaft, Deutsche Krebshilfe, AWMF (Leitlinienprogramm Onkologie 2018)].

Guideline concordant therapy has been shown to improve survival in many entities in real-world cohorts (Wöckel et al. 2010; Inwald et al. 2014; Ahmed et al. 2017; Wimmer et al. 2019; Taubenhansl et al. 2020; Dholakia et al. 2020).

Having acknowledged limited existing data concerning the therapeutic effects of diverse therapeutic modalities in type 2 endometrial cancer, this study sought to address benefits from guideline-concordant therapy. Therefore, we examined the effect on OS and RFS of therapy according to German guidelines issued consecutively over the years.

## Materials and methods

### Database and study design

**Fig. 1:**
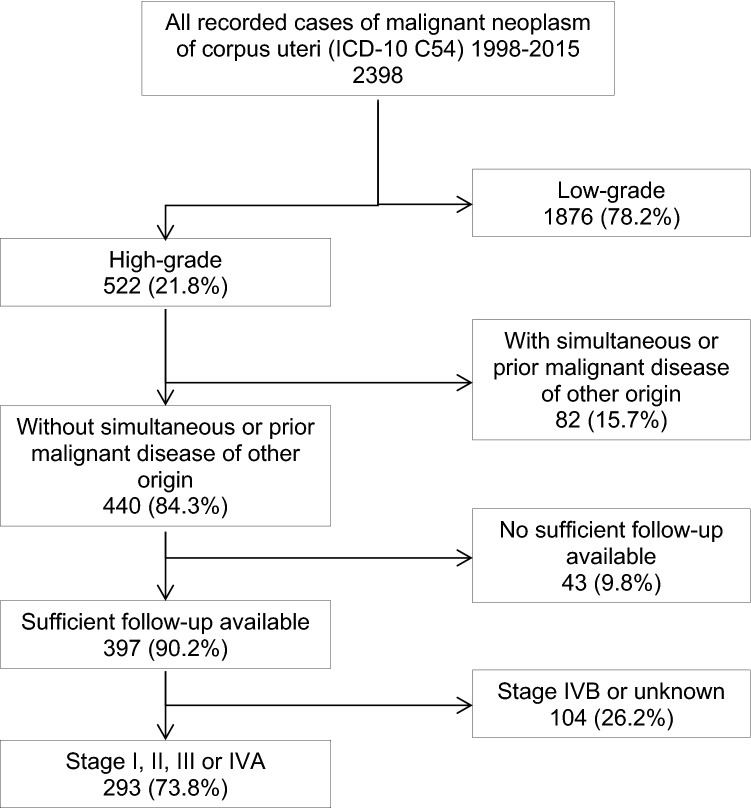
Study population and Inclusion criteria

Our data are derived from the Tumor Center Regensburg (Bavaria, Germany) and has been described previously (Scharl et al. 2018). Patient data from 2398 cases of endometrial cancer (ICD-10 C54) diagnosed between January 1998 and December 2015 in the Region of Upper palatine and Lower Bavaria were reviewed. For this population-based, retrospective cohort study, we selected all patients in the cancer registry who were diagnosed with high-grade endometrial cancer. High-grade endometrial cancer was defined as non-endometrioid histologies, such as serous, clear cell or mixed cell cancer, carcinosarcoma, as well as endometrioid cancer grade 3. Exclusion criteria were previous or synchronous secondary tumors, FIGO stage IVB, unknown tumor stage, and insufficient follow-up. This led to a cohort of 293 patients (Fig. 1).

### Guideline conformity

For each patient in the cohort, we assessed whether the treatment she received corresponds to the treatment recommended by the German S2k or S3 guidelines published in 2008 and 2018, respectively [Deutsche Krebsgesellschaft, Deutsche Krebshilfe, AWMF (Leitlinienprogramm Onkologie 2018)]. The treatment was recorded as guideline-conform treatment (GCT) for S2k or S3 guidelines irrespective of whether the guideline was already available at the time of treatment. Both guidelines recommend hysterectomy, bilateral salingovarectomy, and systematic pelvic and paraaortic lymphadenectomy for FIGO stages I–III; for stage IVA, surgery aiming for macroscopic tumor clearance (i.e., exenteratio) with systematic lymphadenectomy is recommended. In the S2k guideline, systematic lymphadenectomy is defined as the removal of at least 15 pelvic and 10 paraaortic lymph nodes, and the current S3 guideline merely requires an adequate number of pelvic and paraaortic lymph nodes.

The main difference between the guidelines, however, is in the field of radiotherapy and chemotherapy. While the S2k guideline requires either teletherapy or chemotherapy in high-grade histologies or stage III/IVA tumors, the S3 guideline recommends both treatment modalities for stage III and IV tumors. In early stages with high-grade histologies (pT1b/2 G3), radiotherapy in the form of brachytherapy is required and chemotherapy may be performed according to the S3 guideline.

### Statistical evaluation

Continuous data were expressed as means, median, range, and standard deviation (SD). Categorical data were expressed as frequency counts and percentages. Comparison of means was performed by Student’s t test in case of normal distribution; otherwise, Mann–Whitney *U* test was applied. Chi-square test was used for comparisons of categorical variables. Overall survival (OS) was calculated from the date of cancer diagnosis to the date of death from any cause. Recurrence-free survival (RFS) and cumulative recurrence rates included local, regional, and distant relapses. Risk adjustment was performed in multivariable Cox-regression analyses to adjust for confounding factors. The following factors were tested in univariate analysis: age at diagnosis, year of diagnosis, comorbidity, obesity, lymph vessel invasion, blood vessel invasion, Charlson Comorbidity Index (CCI) (Charlson et al. 1987), FIGO stage, and histological subtype. Only factors significant in univariate analysis were included in the multivariate model. Hazard ratios (HR) were considered significant if the corresponding confidence interval (CI) excluded 1, and the p value of the log-rank test was < 0.05. All analyses were performed using IBM SPSS Statistics Version 25.0 (Chicago, EUA).

## Results

### Patient characteristics and clinical interventions

293 patients matched the selection criteria for this study (Fig. 1). All patients were diagnosed with high-grade endometrial carcinoma FIGO stage I-IVA. The median age of patients selected for this study was 69.6 years (range 36–92 years, SD 9.2).

The mean follow-up time in the cohort was 9.46 years (SD 0.36).

Eleven patients displayed a premenopausal (3.8%) and five displayed a perimenopausal status (1.7%), whereas the overwhelming majority of 209 patients (71.3%) were classified as postmenopausal.

172 of 293 (58.7%) patients displayed cancer of FIGO stage I, 29 (9.9%) displayed FIGO stage II, 87 patients (29.7%) were diagnosed with FIGO stage III, and 5 patients (1.7%) with FIGO stage IVA. The majority of patients (*n* = 201; 68. 6%) was diagnosed with type 1 G3 cancer. 63 patients (21.5%) were diagnosed with type 2 endometrial carcinoma and 29 patients (9.9%) with carcinosarcoma. Approximately three-quarters of the selected patients (*n* = 218, 74.4%) suffered from no relevant morbidity other than endometrial cancer (Charlson Comorbidity score = 2), 75 (25.6%) displayed a CCI > 2. Lymph vessel invasion was present in 76 patients (25.9%), and blood vessel invasion in 32 patients (10.9%). Table 1 summarizes the demographic and clinical characteristics of this study group.

**Table 1:** Characteristics of patients treated according to S3 guideline

	S3-guideline-concordant therapy						Chi-square *p*
	No		Yes		Total		
	*n*	%	*n*	%	*n*	%	
Age at date of diagnosis							
< 60	27	15.0	22	19.5	49	16.7%	< 0.001*
60–69	51	28.3	53	46.9	104	35.5%	
70–79	82	45.6	32	17.8	114	38.9%	
80 +	20	11.1	6	5.3	26	8.9%	
Menopausal status							
Premenopausal	7	3.8	4	3.5	11	3.8%	0.780
Perimenopausal	2	1.1	3	2.7	5	1.7%	
Postmenopausal	128	71.1	81	71.6	209	71.3%	
Unknown	43	23.8	25	22.1	68	23.5%	
Year of diagnosis							
1998–2003	47	26.1	18	15.9	65	22.2%	< 0.001*
2004–2009	69	38.3	27	23.8	96	32.8%	
2010–2015	64	35.5	68	60.2	132	45.1%	
Histologic subtype							
Type 1	113	62.7	88	77.9	201	68.6%	0.025*
Carcinosarcoma	21	11.7	8	7.1	29	9.9%	
Type 2	46	25.6	17	15.0	63	21.5%	
CCI							
2	135	75.0	83	73.5	218	74.41%	0.767
> 2	45	25.0	30	26.5	75	25.6%	
FIGO stage							
I	86	47.8	86	76.1	172	58.7%	< 0.001
II	17	9.4	12	10.6	29	9.9%	
III	72	40.0	15	13.3	87	29.7%	
IVA	5	2.8	0	0	5	1.7%	
Gesamt	291	100.0	113	100.0	293	100.0%	

Significant differences were labeled with*

### S2k-guideline adherent therapy

Total concordance with S2k guidelines was 16.0% (*n* = 47). Recommendations regarding chemotherapy, radiotherapy, and surgery were adhered to in 77.5, 56.7, and 38.9%, respectively. Guideline concordant surgery to the primary tumor, lymph nodes, and omentum was performed in 91.5, 35.3, and 89.4%, respectively.

The 5-year OS was 67.9 and 56.3% for patients treated in adherence and non-adherence to the S2k guideline, respectively. Adherence to the S2k guideline published in 2008 did not lead to a significant increase in OS in the complete cohort (*p* = 0.105), nor in early stage cancer (*p* = 0.105). Factors significantly influencing OS in univariate analysis were histological subtype, age, FIGO stage, CCI, lymph vessel invasion, and obesity (Table 2).

**Table 2:** Effect of S3 GCT on OS in patients with high-grade endometrial carcinoma: Results of univariable and multivariable Cox-regression analysis

	*N*	Cox regression				Cox regression			
		Univariable				Univariable			
		95% CI				95% CI			
		*p*	HR	Lower	Upper	*p*	HR	Lower	Upper
S3 GCT									
No	180	< 0.001*	1.000			0.018*	1.000		
Yes	113		0.429	0.298	0.617		0.623	0.420	0.923
Age at diagnosis									
< 60	49	< 0.001*	1.000			< 0.001*	1.000		
60–69	104	0.262	1.385	0.784	2.448	0.218	1.444	0.805	2.592
70–79	114	0.001	2.437	1.415	4.195	0.002	2.409	1.365	4.251
80 +	26	< 0.001	5.026	2.627	9.615	< 0.001	5.414	2.705	10.834
Charlson Index									
2	218	0.020*	1.000			0.502	1.000		
> 2	75		1.502	1.065	2.117		1.139	0.779	1.665
Year of diagnosis									
1998–2003	65	0.077	1.000						
2004–2009	96	0.033	1.580	1.037	2.406				
2010–2015	132	0.458	1.184	0.758	1.849				
FIGO stage									
I	172	0.001	1.000			0.013*	1.000		
II	29	0.705	1.116	0.632	1.972	0.318	1.350	0.750	2.430
III/IVA	92	< 0.001	1.990	1.418	2.793	0.003	1.761	1.207	2.568
Obesity									
No	50	0.012*	1.000			0.017*	1.000		
Yes	243		1.645	1.118	2.419		1.607	1.050	2.460
Histological subgroup									
Type I G3	201	0.001*	1.000			< 001*	1.000		
Type II	29	< 0.001	2.453	1.489	4.040	< 0.001	2.752	1.621	4.673
CS	63	0.032	1.525	1.037	2.244	0.076	1.417	0.944	2.216
Lymph vessel invasion									
L0	136	013*	1.000			0.587	1.000		
L1	76	010	1.654	1.126	2.430	0.332	1.228	0.811	1.858
LX/ns	81	850	0.963	0.648	1.430	0.947	1.014	0.669	1.538
Vein invasion									
V0	168	0.219	1.000						
V1	32	0.124	1.496	0.895	2.499				
VX/ns	93	0.689	0.929	0.649	1.330				

5-Year RFS for patients treated according to the S2k guideline was 66.0% versus 50.3% for patients not treated S2k guideline adherent. A trend toward an improvement on RFS was observed for S2k guideline-concordant treatment in the complete cohort (*p* = 0.070) and early stages (*p* = 0.074). Factors significantly influencing RFS in univariate analysis were histological subtype (*p* < 0.001), age (*p* < 0.001), FIGO stage (*p* < 0.001), lymph vessel invasion (*p* = 0.003), blood vessel invasion (*p* = 0.039), and obesity (*p* = 0.006).

The 5-year recurrence rates for patients treated according to the S2k guideline were 26.1% compared to 31.3% for patients not treated according to the S2k guideline, respectively (*p* = 0.517). The recurrence rate was significantly influenced by FIGO stage (*p* = 0.002) lymph vessel invasion (*p* < 0.001), blood vessel invasion (*p* = 0.001), and histological subtype (*p* = 0.010).

A multivariate analysis yielded similar results. Treatment concordant with the S2k guideline did not result in a significantly higher OS in the complete cohort (HR 0.783, CI 0.465–1.316, *p* = 0.335) or early stage cancer (HR 1.032, CI 0.459–2.322, *p* = 0.939).

RFS was not significantly improved by S2k guideline-concordant therapy in the complete cohort (HR 0.741, CI 0.347–1.740, *p* = 0.242) nor in early stages (HR 0.777, CI 0.131–1.111, *p* = 0.540).

The recurrence rate was not influenced by S2k guideline-concordant therapy in the complete cohort (HR 0.760, CI 0.339–1.446, *p* = 0.403) nor in early stages (HR 0.439, CI 0.133–1.456, *p* = 0.178).

### S3-guideline adherent therapy

113 patients (38.6%) were treated adherent to the S3 guideline. Recommendations regarding chemotherapy, radiotherapy, and surgery were adhered to in 76.5, 70.2, and 54.9%, respectively.

Guideline concordant surgery to the primary tumor, lymph nodes, and omentum was performed in 87.7, 63.1, and 94.8%, respectively.

**Fig. 2:**
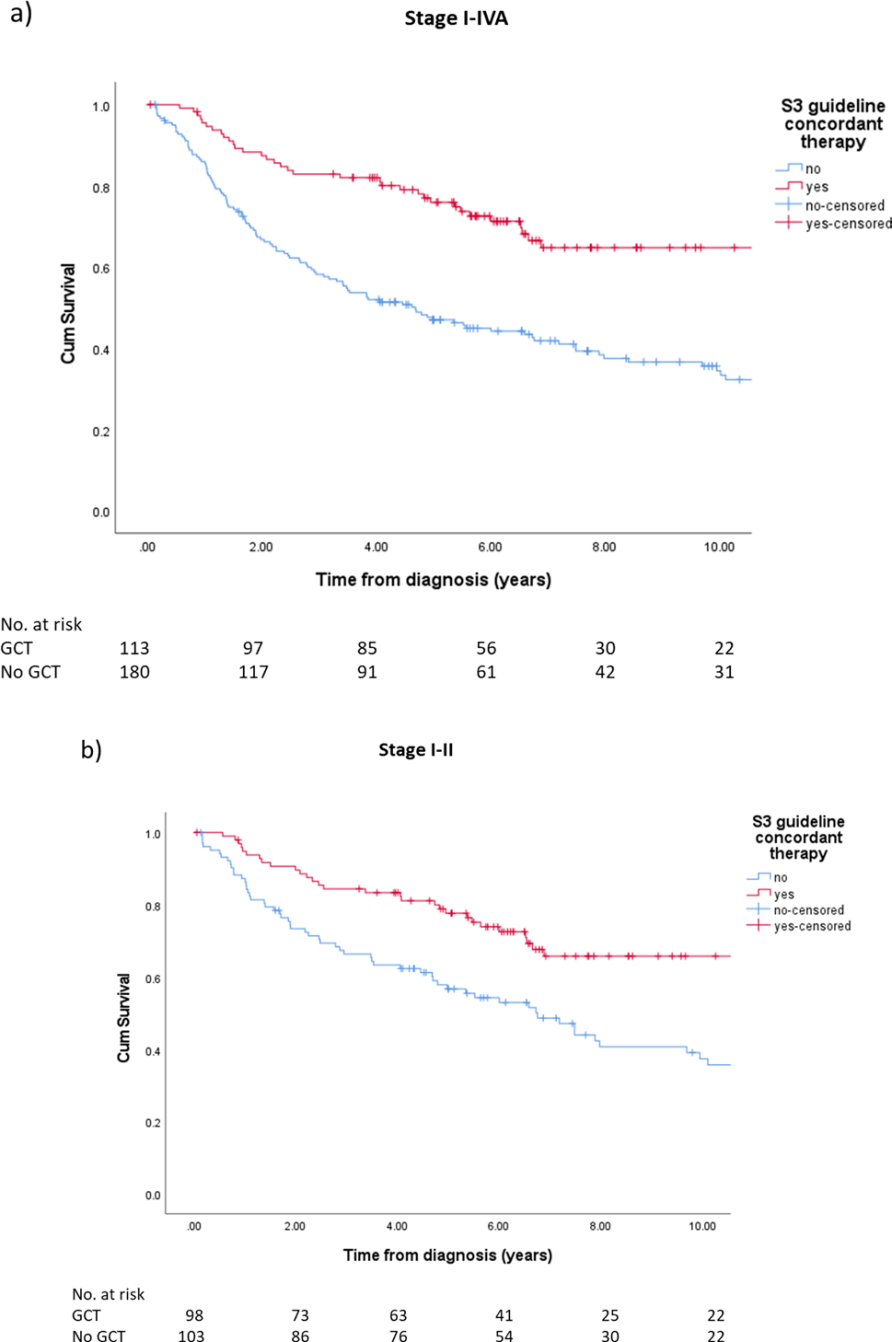
Overall survival in endometrial cancer patients according to guideline-concordant therapy for the whole cohort (**a**) and early stages (**b**)

5-Year OS was 75.9% for patients treated adherent to the S3 guideline compared to 47.0% for those not treated accordingly. Treatment according to the current S3 guideline resulted in a highly significant improvement of OS in the complete cohort when using Kaplan–Meier-Method (*p* < 0.001). A similar result was obtained for early stage cancer (FIGO stage I–II) (*p* < 0.001) (Fig. 2).

**Fig. 3:**
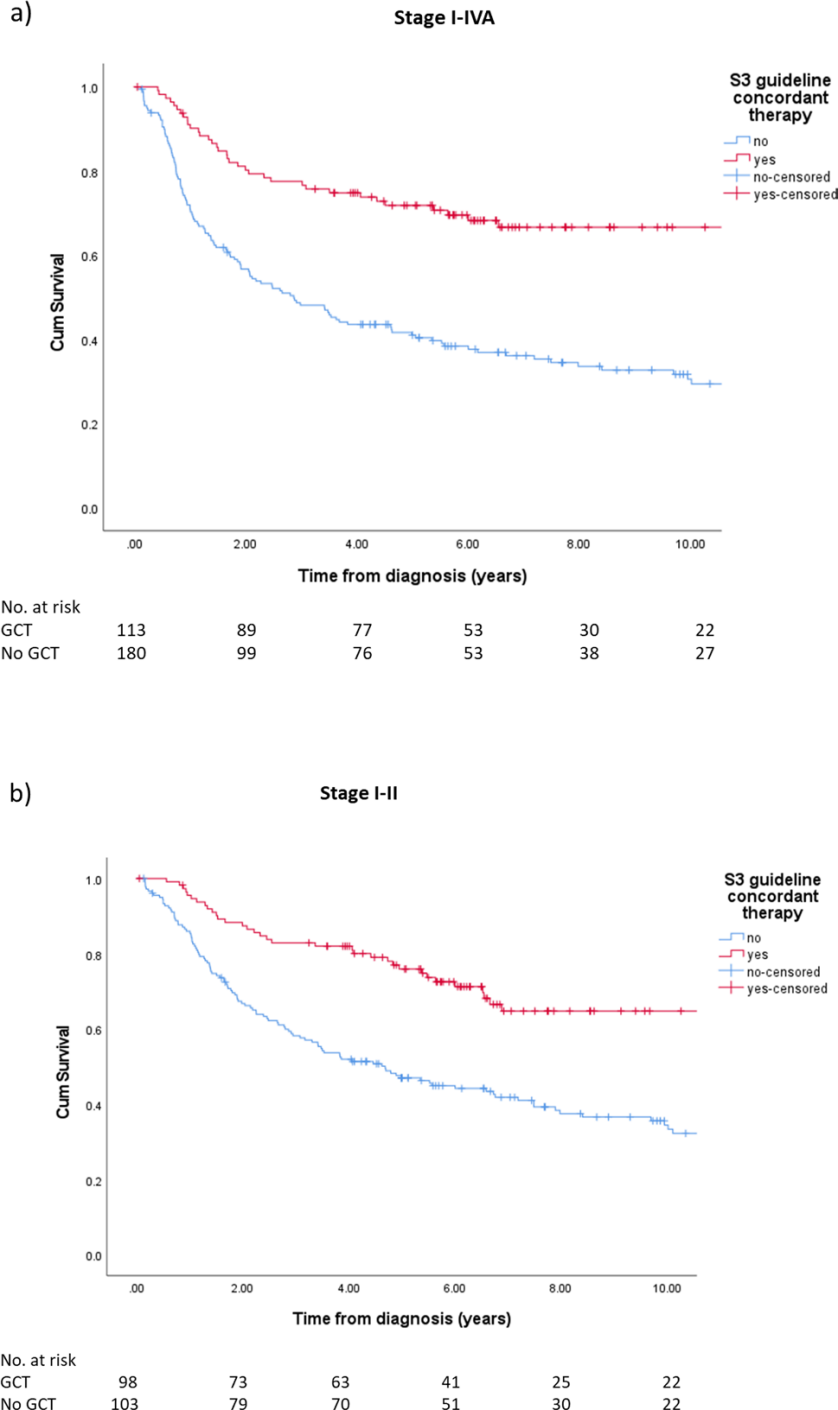
Recurrence-free survival in endometrial cancer patients according to guideline-concordant therapy for the whole cohort (**a**) and early stages (**b**)

5-Year RFS was 71.8 and 41.0% in patients treated S3 guideline adherent and non-adherent, respectively. RFS was improved by treatment according to S3 guideline in the complete cohort (*p* < 0.001) and in early stage cancer (*p* < 0.01) (Fig. 3).

**Fig. 4:**
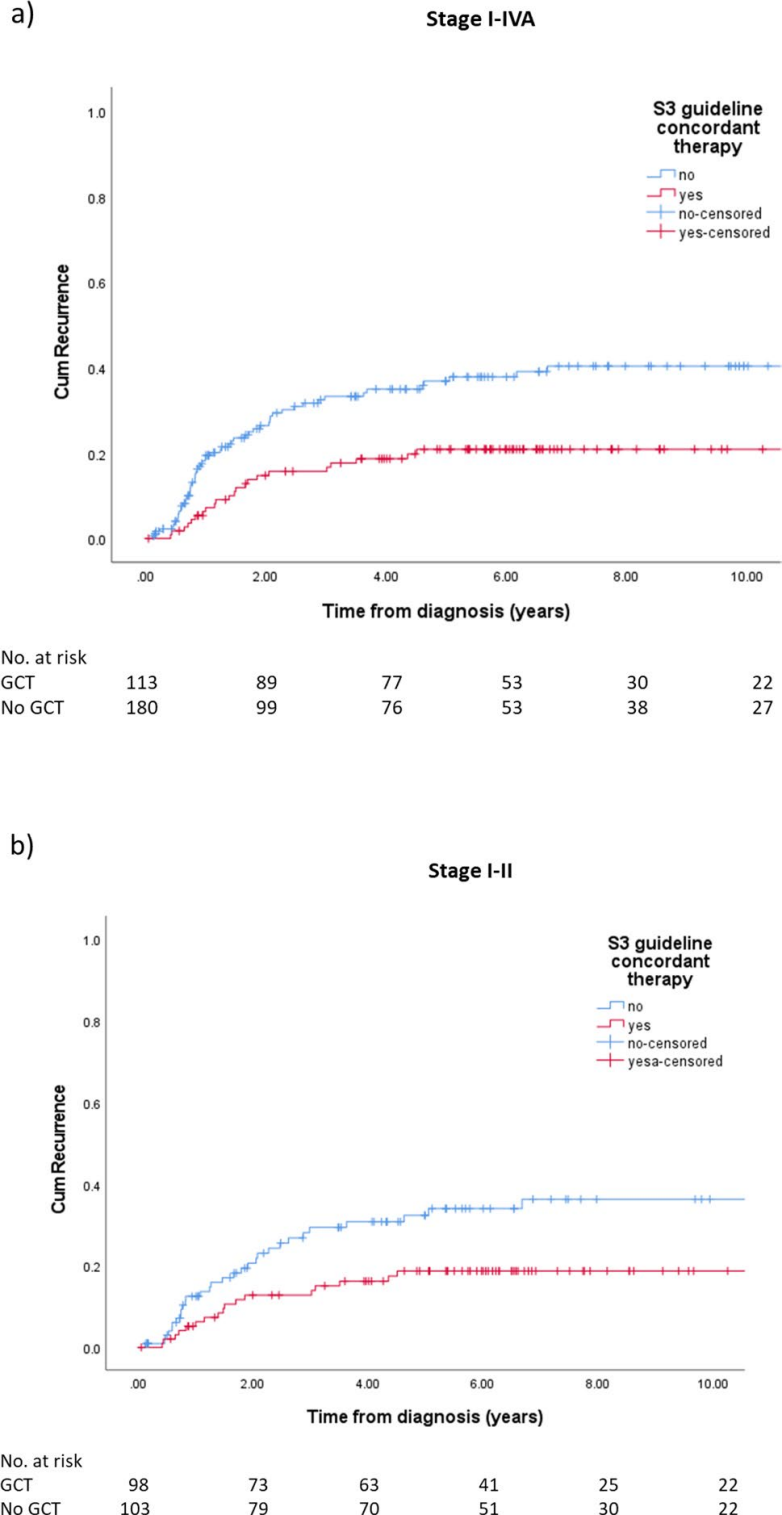
Recurrences in endometrial cancer patients depending on S3 guideline-concordant therapy for the whole cohort (**a**) and early stages (**b**)

The cumulative 5-year recurrence rates were significantly lower in patients treated according to S3 guideline (20.9% versus 36.8%, *p* = 0.002, Fig. 4). This was also the case in early stage disease (*p* = 0.014).

In multivariable analysis, treatment concordant with the S3 guideline significantly improved OS in the complete cohort (HR 0.623, CI 0.420–0.923, *p* = 0.018) and borderline significantly in early stage cancer (HR 0.642, CI 0.411–1.001, *p* = 0. 051). Other factors that remained significant in multivariate analysis were histological subtype, age, FIGO stage, and obesity (Table 2).

RFS was improved through S3 guideline concordat therapy in all stages (HR 0.578, CI 0.387–0.863, *p* = 0.007) and early stages (HR 0.561, CI 0.354–0.888, *p* = 0.014). Factors significantly influencing RFS in multivariate analysis were histological subtype (*p* < 0.001), age (*p* < 0.001), FIGO stage (*p* = 0.004), and obesity (*p* = 0.002).

In a multivariate model, treatment according to S3 guideline remained significantly associated with reduced recurrence rates in early stages (HR 0.495, CI 0.253–0.965, *p* = 0.039). For the complete cohort, only a trend toward reduced recurrence rates remained (HR 0.594, CI 0.348–1.016, *p* = 0.057).

## Discussion

We performed a population-based study on 293 high-grade endometrial cancer patients FIGO stage I–IVA to evaluate the effect of S3 guideline-concordant therapy on survival and recurrences. Patients treated accordingly had significantly higher OS and RFS rates and lower recurrence rates. Since high-quality data on high-grade endometrial cancer patients are scarce, many recommendations in the S3 guideline were based on either case series or expert consensus. To our best knowledge, this is the first study demonstrating a survival benefit for high-grade endometrial cancer patients treated according to the current German S3 guideline. In contrast to the carefully selected cohort represented by randomized trials, our cohort is an unselected real-world collective including elderly and comorbid patients. This analysis, therefore, confirms that high-grade endometrial cancer patients benefit from guideline-concordant therapy.

The concordance to the former S2k guidelines and its effect on OS and RFS was also evaluated for comparison. OS and RFS were consistently higher for S3 than for S2k guideline concordance. This suggests a meaningful clinical effect for the changes made during guideline revision. Even though the OS and RFS rates for patients treated according to S2k guidelines were higher than for those not treated according to the guideline, the survival benefit was not significant. The lack of prognostic effect of S2 guideline adherence may be due to low case numbers as only 16% of patients were treated according to S2k guideline recommendations. One reason for lower numbers of treatment adherence to the S2k guideline is that systematic LND requires the removal of at least 15 pelvic and 10 paraaortic lymph nodes in the S2k guideline, while for the S3 guideline, no specific number of lymph nodes is mentioned. Another major difference between the guidelines is the more frequent indication of chemotherapy in high-grade endometrial cancer in the S3 guideline. IN the S2k guideline chemotherapy was not a mandatory component of treatment but used as an optional addition or alternative to radiotherapy in type II cancer or Stage III or IVA (Kommission Uterus der Arbeitsgemeinschaft Gynäkologische Onkologie e.V. in der Deutschen Gesellschaft für Gynäkologie und Geburtshilfe e.V. sowie in der Deutschen Krebsgesellschaft e. V. 2010; Leitlinienprogramm Onkologie (Deutsche Krebsgesellschaft, Deutsche Krebshilfe, and AWMF) 2018).

The positive effect of guideline-concordant therapy has been demonstrated for numerous other entities and for different national guidelines. Guideline concordant therapy was shown to improve OS for NSCLC (Ahmed et al. 2017) and for breast cancer patients in general (Wöckel et al. 2010; Wolters et al. 2015), as well as for specific subtypes like Her2-positive breast cancer and triple-negative breast cancer (Schwentner et al. 2012; Inwald et al. 2014) and recommended adjuvant radiotherapy and chemotherapy (Wimmer et al. 2019; Taubenhansl et al. 2020).

A study among 629 endometrial cancer patients of all histological subtypes and stages showed a significant improvement of OS in the overall study population treated according to the NCCN guideline (Felix et al. 2020). An analysis of different subpopulations revealed a significant improvement for stage IA, low-grade endometrioid, Stage IVA and IVB as well as all endometrioid endometrial cancer patients of all stages. Survival among stage IB and IC both low-grade endometrioid histology was improved with borderline significance. No significant improvement was observed for high-grade endometrioid and non-endometrioid patients (Felix et al. 2020). Moreover, an NCDB study on non-endometrioid endometrial cancer patients showed an improved survival for patients treated according to the NCCN guideline for all races except for Asian/Pacific islanders (Dholakia et al. 2020). The NCCN recommendations consulted for the aforementioned study were the guidelines valid at the time of treatment of each patient. Due to periodic revisions of the NCCN guidelines, the treatment regiments defined as guideline concordant were not consistent over the study period. The current NCCN guidelines differ from the German S3 guideline in several aspects: While sentinel lymph-node biopsy is not recommended in the current S3 guideline, it is the preferred choice in the NCCN guideline. The recommendation regarding sentinel lymph-node biopsy is currently under reconsideration in the consultative paper for the S3 guideline revision. Systematic lymphadenectomy in the German guideline is defined as pelvic and paraaortic LND, based on the NCCN guideline paraaortic LND is optional. The recommendations on chemotherapy are very similar between the NCCN guideline and the German S3 guidelines. Regarding radiotherapy, the NCCN guideline is more lenient toward EBRT in type II endometrial cancer (National Comprehensive Cancer Network 2022).

Certain shortcomings of this study need to be addressed. Due to the retrospective character of the study, a selection bias toward more aggressive treatment for younger and fitter patients is likely, which we tried to cope with by including age and comorbidity in the risk adjustment. This tendency toward a selection bias may be augmented by the fact that the guidelines tested in this study were not actually available at the time of treatment as the S3 guideline was first introduced in 2018, while this study included only patients until 2015.

## Conclusion

Therapy according to the German S3 guideline improved OS and RFS in univariate as well as multivariate analyses in this cohort of high-grade endometrial cancer patients. The benefit of GCT on recurrence rates was significant in univariate analysis. In multivariate analysis, only borderline significance was observed. The benefit of treatment according to the former S2k guideline was not significant. Therefore, an improvement regarding long-time outcome by the guideline revision can be postulated. This study demonstrates that guideline-concordant therapy improves survival in high-grade endometrial cancer patients.

## Data Availability

The datasets generated during and/or analyzed during the current study are available from the corresponding author on reasonable request.
